# Aspirin enhances the sensitivity of hepatocellular carcinoma side population cells to doxorubicin via miR-491/ABCG2

**DOI:** 10.1042/BSR20180854

**Published:** 2018-11-09

**Authors:** Zheng-Yuan Xie, Mao-Sheng Liu, Cong Zhang, Peng-Cheng Cai, Zhi-Hua Xiao, Fen-Fen Wang

**Affiliations:** 1 Department of Gastroenterology, The Second Affiliated Hospital of Nanchang University, Nanchang 330006, China; 2 Department of Gastroenterology, The First Affiliated Hospital, Gannan Medical University, Ganzhou 341000, China

**Keywords:** Aspirin (ASA), hepatocellular carcinoma (HCC), side population (SP), doxorubicin (Doxo), miR-491, ATP-binding cassette sub-family G member 2 (ABCG2)

## Abstract

**Objective:** To explore whether aspirin (ASA) enhances the sensitivity of hepatocellular carcinoma (HCC) side population (SP) cells to doxorubicin (Doxo) via miR-491/ATP-binding cassette sub-family G member 2 (ABCG2).

**Methods:** Non-SP and SP cells were isolated from MHCC-97L cell line using flow cytometry analysis and fluorescence-activated cell sorting. Colony formation assay was performed to determine the colony-formation ability of cells. Cell viability of SP cells was determined with the MTT assay. Luciferase reporter assay was applied in confirming the binding between miR-491 and ABCG2.

**Results:** Although the Doxo treatment lowered the colony-formation ability of both non-SP and SP cells, the colony-formation ability of SP cells was 2-fold higher than that of non-SP cells (*P*<0.05). Doxo slightly inhibited the cell viability of SP cells in a concentration-dependent manner; the addition of ASA dramatically enhanced the inhibitory effect of Doxo on SP cell viability in a concentration-dependent manner (*P*<0.05). Compared with non-SP cells, the miR-491 expression was significantly decreased in SP cells, which was significantly reversed by ASA (*P*<0.05). miR-491 directly controlled the ABCG2 expression. In the presence of Doxo, miR-491 inhibitor reduced the inhibitory effect of ASA on the cell viability of SP cells, which was significantly reversed by knockdown of ABCG2 (*P*<0.05).

**Conclusion:** ASA enhanced the sensitivity of SP cells to Doxo via regulating the miR-491/ABCG2 signaling pathway.

## Introduction

Hepatocellular carcinoma (HCC) is one of the leading causes of cancer-related deaths in the world [1]. Chemotherapeutic agents (including doxorubicin [Doxo]) are widely used in the clinical treatment of HCC [2]. However, drug resistance always results in the failure and thus limited use of chemotherapeutic drugs in treating HCC patients [3]. Therefore, enhancing the drug sensitivity of HCC cells is beneficial for the clinical treatment of patients with HCC.

Side population (SP) cell is a special type of tumor stem cell that exists in many solid tumor tissues, including human primary HCC [4–8]. In HCC cell lines, previous studies have also reported the existence of unique SP cells with cancer stem/stem cell properties [9–11]. Compared with non-SP cells, the SP cells showed much stronger anti-apoptotic and proliferative activities [12]. Besides, it was found that the resistance of SP cells to chemotherapy drugs was significantly higher than that of non-SP cells [13,14].

A common cause of drug resistance is that a large number of tumor cells express the ATP-binding cassette (ABC) pump, which causes tumors to have little response to conventional chemotherapy [15–18]. ATP-binding cassette sub-family G member 2 (ABCG2) is the main transport protein that mediates SP phenotype [19,20]. ABCG2 promotes drug resistance, and is a potential cancer stem cell (CSC) marker in HCC. The expression of ABCG2 is closely related to the occurrence, proliferation, drug resistance, and metastasis of tumor. As reported, the up-regulation of ABCG2 enhanced the proliferation, Doxo resistance, migration, and invasion of HCC, which were lowered by the down-regulation of ABCG2 [21]. Hu et al. [22] studied the expression pattern of ABCG2 in HCC, and proved that the expression of ABCG2 endowed HCC cells, especially SP cells, with the efflux capacity, which was modulated by Akt signaling.

It has been reported that aspirin (ASA), a cyclooxygenase inhibitor, promoted growth inhibition and apoptosis of HCC [23]. The ligation of ASA to cisplatin can lead to chemotherapy sensitization, thereby defeating resistance [24]. Recently, the scholars demonstrated that ASA inhibited the acquisition of chemoresistance in breast cancer by disrupting the NFkB–IL6 signaling pathway that was responsible for the generation of CSCs [25]. However, there have been few studies about the underlying mechanism of how ASA suppresses the drug resistance of HCC SP cells.

MiRNAs, a well-known class of small non-coding RNAs, participate in numerous pathophysiological processes [26]. Multiple miRNAs are related to HCC, including miR-491. miR-491 was reported to be related with the CSC-like properties of HCC [27]. Its expression was much lower in poorly differentiated HCC tissues compared with well-differentiated HCC tissues, and miR-491 was negatively associated with CSC-like properties in both cell line and tissue samples of HCC [27]. Bioinformatics analysis (microRNA.org) has shown the binding site of miR-491 in ABCG2. Therefore, in the present study, we investigated whether ASA enhances the sensitivity of HCC SP cells to Doxo via up-regulating miR-491 and down-regulating target gene ABCG2, providing theoretical basis for the clinical application of ASA.

## Materials and methods

### Isolation of non-SP and SP cells from the HCC cell line MHCC-97L

The isolation of non-SP and SP cells from MHCC-97L cell line was conducted as previously reported [22]. Briefly, the adherent cells were dissolved by trypsin and suspended in DMEM medium containing 2% FBS and 10 mM *N*-2-hydroxyethylpiperazine-*N*′-2-ethanesulfonic acid (1 × 10^6^ cells/ml). The cells were then stained by 5 μg/ml Hoechst 33342 (Invitrogen) at 37°C for 1.5 h in the absence or presence of 10 μM fumitremorgin C (FTC). Afterward, the cells were centrifuged at 4°C and resuspended in ice-cold PBS with 2 μg/ml propidium iodide (PI). Then flow cytometry analysis and fluorescence-activated cell sorting were performed to isolate non-SP and SP cells.

### Colony formation assay

To determine the colony-formation ability of MHCC-97L cells with drug treatment, the SP and non-SP cells were sorted in the presence of Doxo (500 ng/ml). The sorted cells were planted in 96-well microplates in triplicate (20 cells/well) and cultured in DMEM medium with 10% FBS. After 2 weeks, the cells were stained with 0.01% crystal violet and the number of colonies was counted under a microscope.

### MTT assay

The cell viability of SP cells was determined with the MTT assay. Briefly, SP cells were planted into 96-well microplates and cultured for 24 h. Then the cells were treated with different concentrations of ASA (0, 1.25, 2.5, and 5 μmol/ml) for 48 h in the presence of Doxo (500 ng/ml) [28]. Afterward, 10  μl of MTT was added to every well and cultured at 37 °C for 2  h in darkness. After the medium was changed to DMSO, and the absorbance at 570  nm was determined.

### Quantitative real-time PCR analysis

Total RNA was extracted using TRIzol reagent (Invitrogen) according to the manufacturer’s instructions. After the purity and concentration were determined, the purified RNA was used to synthesize the first‐strand cDNA using Reverse Transcription Kit (Qiangen, Germany) under the guidance of the manufacturer. The SYBR Green Master Mix (Applied Biosystems, U.S.A.) was used for quantitative real-time PCR (qPCR), which was analyzed on an ABI 7900HT Fast Real-Time PCR System. The relative expression levels of miR-130b-3p, miR-7-5p, miR-491, miR-612, and miR-3650 were normalized to those of U6, and the relative expression levels of ABCG2 were normalized to those of GAPDH. The gene expression was quantified using the comparative *C*_t_ (ΔΔ*C*_t_) approach.

### Western blotting

The radioimmunoprecipitation assay (RIPA) lysis buffer (containing protease and phosphatase inhibitors) was used to extract total proteins from cells. After measuring the protein concentration, we loaded the equal amounts of protein samples to SDS-PAGE. The proteins were then transferred to a PVDF membrane. After being incubated with 5% non-fat milk for 30 min at room temperature, the PVDF membrane was probed with the primary antibodies, anti-ABCG2 (1:20, Abcam), and anti‐β-actin (1:5000, Abcam), at 4°C overnight. After being washed three times, the membrane was incubated with secondary antibody containing horse radish peroxidase (HRP) at room temperature for 2 h. Bands were visualized with ECL (GE Healthcare).

### Cell transfection

The SP cells were seeded in six-well plates. After 24 h, the SP cells were transfected with miR-491 inhibitor, ABCG2 siRNA (siRNA-ABCG2) or negative control using Lipofectamine 2000 (Invitrogen, U.S.A.) according to the protocol of manufacturer. Forty-eight hours after transfection, the cells were collected for determining gene expression and cell viability.

### Luciferase reporter assay

Genomic DNA was isolated from SP cells and used as a template. The wild-type 3′UTR of ABCG2 was inserted into the pGL3-Basic Luciferase Reporter Vector (Promega) to construct the ABCG2-WT plasmid. Then the mutant ABCG2 3′UTR (ABCG2-Mut) plasmid was constructed based on the ABCG2-WT plasmid. The SP cells were cultured in 12-well plates for 24 h, and then transfected with one of the pGL3-based 3′UTR-reporter plasmids together with miR-491 mimic, miR-491 inhibitor, or negative control using Lipofectamine 2000. The cells were collected after 48 h of transfection, and the luciferase activity was measured using Dual-Luciferase Reporter Assay (Promega, U.S.A.) under the guidance of the manufacturer.

### Statistical analysis

Statistical analysis was conducted using SPSS 18.0 software. Student’s *t*-tests or ANOVA (used in cases where there are more than two groups) were performed to compare the mean of groups. All data were presented as mean ± S.D. *P*<0.05 was considered as statistically significant.

## Results

### ASA enhances the sensitivity of SP cells to Doxo

**Figure 1:**
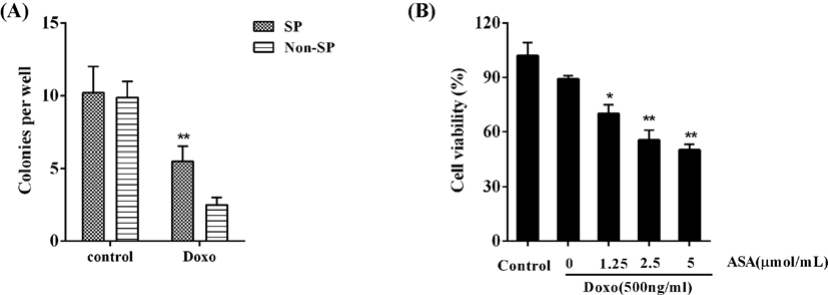
Effect of ASA on drug resistance of HCC SP cells Flow cytometry analysis and fluorescence-activated cell sorting were performed to isolate non-SP and SP cells from the HCC cell line MHCC-97L. (**A**) The colony-formation ability of non-SP and SP cells was determined in the absence or presence of Doxo (500 ng/ml); ^**^P<0.01, compared with Non-SP cells. (**B**) The cell viability of SP cells treated with different concentrations of ASA (0, 1.25, 2.5, and 5 μmol/ml) for 48 h in the presence of Doxo (500 ng/ml) was analyzed by MTT assay; ^*^P<0.05, ^**^P<0.01, compared with control.

Without drug treatment, non-SP and SP cells showed the equivalent colony-formation ability. Although the Doxo treatment lowered the colony-formation ability of both non-SP and SP cells, the colony-formation ability of SP cells was much higher than that of non-SP cells (*P*<0.01; Figure 1A). We then focussed on studying the SP cells. Doxo slightly inhibited the cell viability of SP cells; the addition of ASA dramatically enhanced the inhibitory effect of Doxo on SP cell viability in a concentration-dependent manner (*P*<0.05; Figure 1B).

### Decreased miR-491 expression in SP cells is reversed by ASA

**Figure 2:**
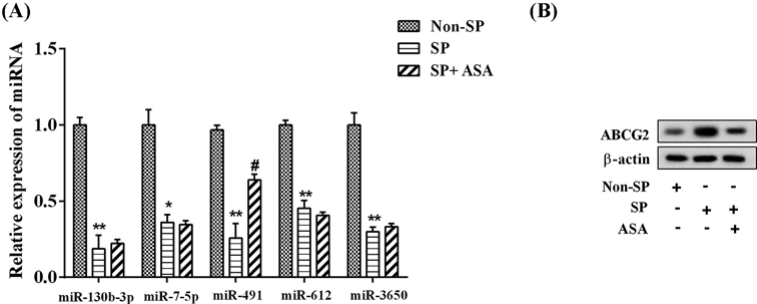
Decreased miR-491 expression in SP cells was reversed by ASA ASA (2.5 μmol/ml) was used to treat MHCC-97L SP cells. There were three groups as follows: non-SP group, SP group, and SP + ASA group. (**A**) The expression of multiple miRNAs (miR-130b-3p, miR-7-5p, miR-491, miR-612, and miR-3650), confirmed by qPCR; ^*^P<0.05, ^**^P<0.01, compared with non-SP cells; ^#^*P*<0.05, compared with SP. (**B**) The expression of ABCG2, determined by Western blotting. β-Actin was used as the loading control.

Compared with non-SP cells, the expression of miR-130b-3p, miR-7-5p, miR-491, miR-612, and miR-3650 was significantly decreased in SP cells; however, ASA can only reverse the miR-491 expression (*P*<0.05; Figure 2A). Consistent with this finding, the ABCG2 protein expression in SP cells was much higher than that in non-SP cells, which was reversed by ASA treatment (Figure 2B).

### ASA enhances the sensitivity of SP cells to Doxo via miR-491

**Figure 3:**
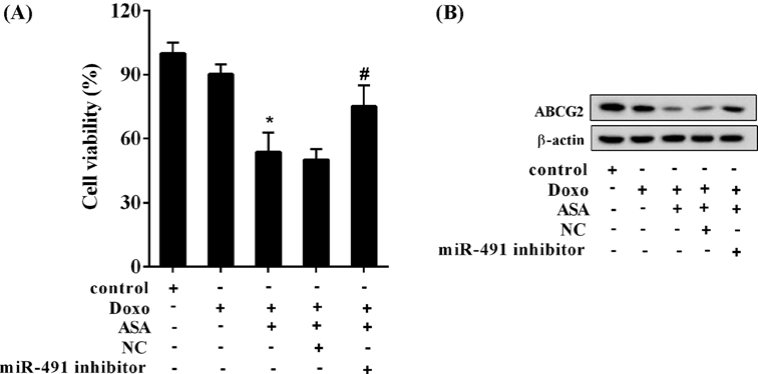
ASA enhanced the sensitivity of SP cells to Doxo via miR-491 The SP cells were divided into five groups (Control, Doxo, Doxo + ASA, Doxo + ASA + NC, and Doxo + ASA + miR-491 inhibitor). The concentration of Doxo was 500 ng/ml, and ASA was 2.5 μmol/ml. (**A**) The cell viability of SP cells; ^*^P<0.05, compared with Doxo; ^#^*P*<0.05, compared with Doxo + ASA + NC. (**B**) The expression of ABCG2.

In the presence of Doxo, the cell viability of SP cells was 90%, which was dramatically decreased by ASA with a result of 55%, and further significantly reversed by the miR-491 inhibitor with a result of 72% (*P*<0.05; Figure 3A). In line with this finding, the ABCG2 expression was reduced by ASA, which was reversed by the miR-491 inhibitor (Figure 3B).

### miR-491 directly controls the ABCG2 expression

**Figure 4:**
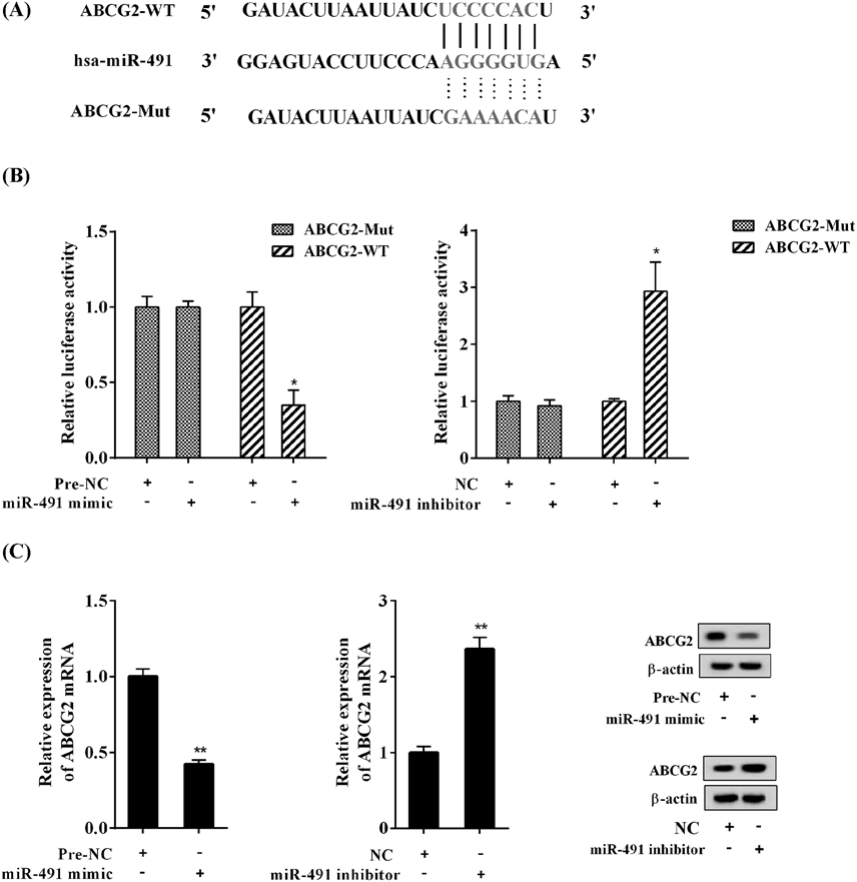
miR-491 directly controlled the ABCG2 expression in SP cells (**A**) The miR-491-binding site existed in the 3′UTR of ABCG2 mRNA. (**B**) The effect of miR-491 mimic or inhibitor on the luciferase activity of wild-type ABCG2 3′UTR and mutant ABCG2 3′UTR. (**C**) The effect of miR-491 mimic or inhibitor on the mRNA and protein expression of ABCG2. ^*^P<0.05 and ^**^P<0.01, compared with pre-NC or NC.

The bioinformatics analysis showed the miR-491-binding site in the 3′UTR of ABCG2 mRNA (Figure 4A). In SP cells, the luciferase reporter assay showed that miR-491 mimic inhibited the luciferase activity of wild-type ABCG2 3′UTR (*P*<0.05), without affecting the mutant ABCG2 3′UTR; in contrast, miR-491 inhibitor enhanced the luciferase activity of wild-type ABCG2 3′UTR (*P*<0.05), without impacting the mutant ABCG2 3′UTR (Figure 4B). Further, miR-491 mimic reduced the mRNA and protein expression of ABCG2, while miR-491 inhibitor increased the mRNA and protein expression of ABCG2 (Figure 4C).

### ASA enhances the sensitivity of SP cells to Doxo via miR-491/ABCG2

**Figure 5:**
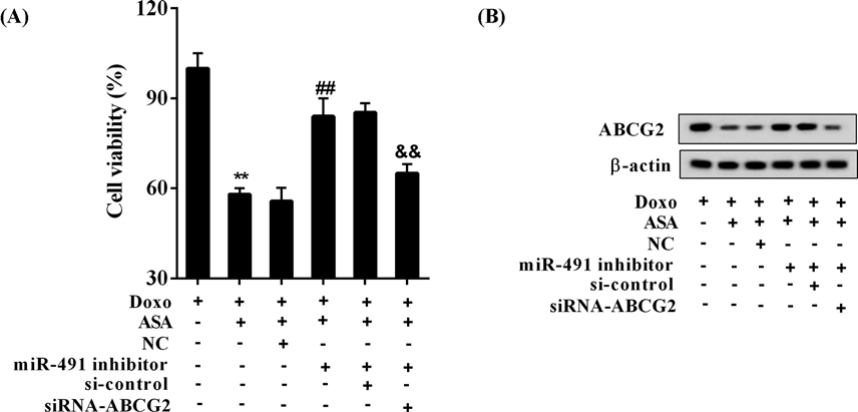
ASA enhanced the sensitivity of SP cells to Doxo via miR-491/ABCG2 The SP cells were allocated to five groups (Doxo, Doxo + ASA, Doxo + ASA + NC, Doxo + ASA + miR-491 inhibitor, Doxo + ASA + miR-491 inhibitor + si-control, and Doxo + ASA + miR-491 inhibitor + siRNA-ABCG2). The concentration of Doxo was 500 ng/ml, and ASA was 2.5 μmol/ml. (**A**) The cell viability of SP cells; ^**^P<0.01, compared with Doxo; ^##^*P*<0.01, compared with Doxo + ASA + NC; ^&&^*P*<0.01, compared with Doxo + ASA + miR-491 inhibitor + si-control. (**B**) The protein expression of ABCG2 in SP cells.

In the presence of Doxo, miR-491 inhibitor reduced the inhibitory effect of ASA on the cell viability of SP cells, which was significantly reversed by the knockdown of ABCG2 (*P*<0.05; Figure 5A). Consistent with this finding, the promoting effect of ASA on ABCG2 expression was decreased by miR-491 inhibitor, which was reversed by the knockdown of ABCG2 (Figure 5B).

## Discussion

In the present study, we found that the colony-formation ability of SP cells were much higher than that of non-SP cells, and that ASA enhanced the sensitivity of SP cells to Doxo via up-regulating miR-491 and down-regulating target gene ABCG2.

Chemotherapy is one of the main methods for clinical treatment of HCC patients [1]. However, the use of chemotherapeutic drugs, including Doxo and cisplatin, was limited due to the drug toxicity, low drug efficacy, and acquired drug resistance of cancer cells [29]. Due to the high incidence of developing resistance to drug in HCC [3], it is important to enhance the drug sensitivity of HCC cells in clinical treatment.

SP cell is a special type of tumor stem cell that has been isolated from multiple solid tumor tissues, such as small-cell lung cancer (SCLC), cervical cancer, osteosarcoma, and HCC [4–7,30,31]. In human primary HCC, the cell viability, colony forming ability, anti-apoptosis, self-renewal, invasion, and tumorigenicity of SP cells were much higher than those of non-SP cells [32]. Moreover, it has also been reported that the resistance of SP cells to chemotherapy drugs was significantly higher than that of non-SP cells [9,10,13,14]. Consistent with this, in the present study, we found that the colony-formation ability of SP cells were much higher than that of non-SP cells, although the Doxo treatment lowered the colony-formation ability of both non-SP and SP cells. Besides, Doxo can only slightly suppress the cell viability of SP cells. As reported, ABCG2 promoted the drug resistance, and was a potential CSC marker in HCC [21]. Moreover, the expression of ABCG2 endowed HCC cells, especially SP cells, with the efflux capacity. In our study, we also found that the ABCG2 expression in SP cells was much higher than that in non-SP cells.

To date, the chemotherapy of HCC is widely accepted in the world. For example, Doxo is widely used in Asia and the North-African region; Sorafenib is the most popular drug for the HCC treatment worldwide, new PD1-inhibitors and regorafenib are also recently approved. ASA is widely applied in the chemotherapy of HCC owing to its anti-platelet effect [33]. ASA was found to enhance IFN-α-induced growth inhibition and apoptosis of HCC via controlling the Janus kinase 1 (JAK1)/signal transducer and activator of transcription 1 (STAT1) signaling pathway [23]. Subsequently, researchers demonstrated that the ligation of ASA to cisplatin could take significant synergistic effects on tumor cells [24]. Recently, it has been reported that ASA inhibited the acquisition of chemoresistance in breast cancer by disrupting the NFkB–IL6 regulatory axis that contributed to the generation of CSCs [25]. In the present study, we explored the underlying mechanism of how ASA suppressed the drug resistance of HCC SP cells. miR-491 is widely involved in the pathogenesis of multiple tumors, including glioma, osteosarcoma, cervical cancer, esophageal cancer, and liver cancer by regulating cell proliferation, apoptosis, migration, invasion, etc [34–38]. In HCC, miR‐491 was shown to be involved in metastasis by blocking epithelial-to-mesenchymal transition (EMT) and reducing matrix metalloproteinase (MMP)‐9 expression [39]. Besides, miR-491 was shown to decrease CSC-like properties of HCC by inhibition of GIT-1/NFκB-mediated EMT [27]. In the present study, we found that the miR-491 expression was significantly decreased in SP cells compared with non-SP cells, which was significantly reversed by ASA. Moreover, miR-491 directly controlled the ABCG2 expression. In the presence of Doxo, miR-491 inhibitor reduced the inhibitory effect of ASA on the cell viability of SP cells, which was significantly reversed by knockdown of ABCG2. Therefore, ASA enhanced the sensitivity of HCC SP cells to Doxo via up-regulating miR-491 and down-regulating target gene ABCG2. However, the ASA/miR-491/ABCG2 regulatory axis was not confirmed *in vivo*, requiring further investigations.

In conclusion, ASA enhanced the sensitivity of SP cells to Doxo via regulating the miR-491/ABCG2 signaling pathway, providing theoretical basis for the clinical application of ASA.

## Abbreviations

ABC: ATP-binding cassette
ABCG2: ATP-binding cassette sub-family G member 2
ASA: aspirin
CSC: cancer stem cell
DMEM: Dulbecco’s modified Eagle medium
Doxo: doxorubicin
EMT: epithelial-to-mesenchymal transition
FTC: fumitremorgin C
HCC: hepatocellular carcinoma
JAK1: Janus kinase 1
NC: negative control
PI: propidium iodide
RIPA: radioimmunoprecipitation assay
SCLC: small-cell lung cancer
SP: side population
STAT1: signal transducer and activator of transcription 1
WT: wild type
